# A novel mechanism by which c-MYC is aberrantly activated by epigenetic silencing of its antisense lncRNA in colon cancer

**DOI:** 10.3389/fgene.2025.1552009

**Published:** 2025-10-21

**Authors:** Xuming Hu, Ye Wei, Meiying Zhang, Chunfeng Dou, Liping Wang, Gul Zaib, Huixian Wu, Wang Guo, Xiaoyuan Wang, Shihao Chen, Qi Xu, Mingzhou Guo, Hengmi Cui

**Affiliations:** ^1^ Institute of Epigenetics and Epigenomics, Yangzhou University, Yangzhou, Jiangsu, China; ^2^ Joint International Research Laboratory of Agricultural & Agri-Product Safety, Ministry of Education of China, Yangzhou University, Yangzhou, Jiangsu, China; ^3^ Jiangsu Co-Innovation Center for Prevention & Control of Important Animal Infectious Diseases & Zoonoses, Yangzhou, Jiangsu, China; ^4^ Department of Gastroenterology and Hepatology, Chinese PLA General Hospital, Beijing, China; ^5^ Department of General Surgery, The Second Affiliated Hospital of Nanjing Medical University, Nanjing, Jiangsu, China; ^6^ Jiangsu Innovation Institute for Biomedicine, Nanjing, Jiangsu, China

**Keywords:** antisense RNA, c-Myc, DNA methylation, cancer, HuR

## Abstract

**Background:**

Proto-oncogenes are abnormally activated in nearly all types of tumors. However, the epigenetic mechanism of proto-oncogene activation has not yet been well elucidated.

**Methods:**

The present study involved the construction of a double-stranded cDNA library derived from gastrointestinal cancer cells, followed by high-throughput genome sequencing to select the c-MYC gene associated with colorectal cancer. Through RACE analysis, we identified the antisense RNA MYC-AS1 and its complete sequence. By investigating the cellular functions, expression of MYC-AS1, elucidating its interaction mechanism with the c-MYC gene, and exploring the impact of DNA methylation on MYC-AS1 expression, we uncover the fundamental principles and regulatory mechanisms underlying colorectal cancer development.

**Results:**

Here, we show that a subset of proto-oncogenes,including c-MYC, possess antisense RNAs. Upregulation of c-MYC in cancer tissues was attributed to the silencing of its antisense RNA MYC-AS1 via DNA hypermethylation. MYC-AS1 RNA markedly inhibited the proliferation of cancer cells in vitro and impeded tumor growth in nude mice in vivo by repressing the expression of c-MYC via an RNAi mechanism. MYC-AS1 RNA bound directly to the HuR protein in the cytoplasm, enhancing the RNA stability of MYC-AS1. Furthermore, MYC-AS1 inhibited c-MYC-targeted gene LDHA expression. Unlike the well-characterized oncogenic long noncoding RNA PVT1, which is coamplified with MYC and enhances its stability, MYC-AS1 is epigenetically silenced and functions as a tumor suppressor through an RNAi mechanism, revealing a distinct layer of MYC regulation.

**Conclusion:**

Our work provides a novel mechanism by which c-MYC is activated in cancer cells by epigenetic silencing of its antisense RNA, which functions as a tumor suppressor.

## Statement of significance

The present study reveals a novel mechanism of tumorigenesis by which oncogenes are activated by epigenetic silencing of their counterpart antisense lncRNAs. Identifying MYC-AS1 as a tumor suppressor may facilitate the design of new anticancer drugs.

## Introduction

Abnormal loss of function of tumor suppressor genes (TSGs) and activation of proto-oncogenes are important molecular characteristics in various types of cancer cells. Accumulated evidence shows that genetic mutations and/or epigenetic silencing result in loss of function of TSGs (Feinberg et al., 2006; Mohammad et al., 2019). The discovery of epigenetic silencing of TSGs led to the introduction of epigenetic drugs into the arsenal of clinical cancer therapies (Mohammad et al., 2019). For example, DNA methylation inhibitors, such as 5-Aza-2-deoxycytidine (5-Aza-CdR) and 5-azacytidine (5-Aza-CR), have been approved by the USA Food and Drug Administration (FDA) for the treatment of myelodysplastic syndrome (MDS) (Kaminskas et al., 2005) and are undergoing active investigation for solid tumors (Yang et al., 2014). Although genetic alterations, such as point mutation and amplification, contribute to oncogene activation, the epigenetic mechanisms of proto-oncogene activation remain unclear. In addition, the molecular mechanism and clinical efficacy of DNA methylation inhibitors are under debating (Issa and Kantarjian, 2009). It is unclear whether oncogenes are simultaneously upregulated when TSGs are activated by DNA methylation inhibitors due to their global demethylation.

The well-known proto-oncogene c-MYC contributes to the initiation and maintenance of tumorigenesis in many human cancers (Casey et al., 2016; Dang, 2012; Stine et al., 2015; Topper et al., 2020). Although it is well known that genetic alterations, such as point mutations and amplification, contribute to the activation of c-MYC, the epigenetic mechanism of the proto-oncogene activation has not yet been well elucidated. Recently, several studies revealed that epigenetic drugs, such as the DNA methylation inhibitors 5-aza-2-deoxycytidine (5-aza-CdR) and 5-azacytidine (5-aza-CR), were involved in the epigenetic regulation of the c-MYC gene by inhibiting its protein expression (Topper et al., 2017), but no a rational explanation was given for this phenomenon.

Epigenetic mechanisms, particularly promoter DNA hypomethylation, have been implicated in the activation of certain oncogenes. However, the specific epigenetic pathways responsible for MYC activation in the absence of genetic lesions remain inadequately defined. A largely unexplored dimension of epigenetic control involves long noncoding RNAs (lncRNAs), particularly natural antisense transcripts, which can exert profound effects on gene expression in cis or in trans (Prensner and Chinnaiyan, 2011; Moran et al., 2012). Notably, while the oncogenic potential of the MYC-adjacent lncRNA PVT1 is well-established, the existence and functional significance of a MYC-specific antisense transcript acting as a tumor suppressor have not been elucidated. Here, we identify MYC-AS1 as such a transcript, and demonstrate that its epigenetic silencing via promoter hypermethylation constitutes a fundamental mechanism for MYC activation in cancer, revealing a new class of tumor-suppressive regulation.

In the previous study, we demonstrated that an antisense RNA can induce hypermethylation of its sense tumor suppressor gene (TSG) *p15*, giving rise to epigenetic silencing of the gene in AML (Yu et al., 2008). Consistent with this, the tumor suppressor gene *p21* also possesses an antisense RNA that inhibits the expression of *p21* through epigenetic mechanisms (Morris et al., 2008). These results suggest that antisense RNAs in cancer cells may downregulate tumor suppressor genes in cancer cells by inducing epigenetic modifications. In this study, we unexpectedly found that a set of oncogenes also possess their antisense RNAs by high-throughput RNA-Seq analysis. By studying a well-known oncogene *c-MYC*, we found that epigenetic silencing of its antisense RNA MYC-AS1 directly contributes to activation or upregulation of the c-MYC gene. We further uncovered that the antisense RNA MYC-AS1 functions as a tumor suppressor that strictly controls *c-MYC* expression. Once this suppressor becomes defective due to events such as epigenetic silencing, the oncogene *c-MYC* is abnormally activated and it's like the tumor suppressor brake is released. MYC-AS1 RNA binds directly to HuR protein in the cytoplasm, enhancing the RNA stability of MYC-AS1. Furthermore, MYC-AS1 inhibited c-MYC-targeted gene LDHA expression. These results reveal a novel mechanism of tumorigenesis by which the oncogene is activated by epigenetic silencing of its antisense lncRNA.

## Experimental procedures

### Human tissue samples

Surgical resection specimens including cancer tissues and matched normal tissues were obtained from the Chinese PLA General Hospital in Beijing. A total of 50 pairs from colon cancer patients were collected for expression analysis. The collection of all specimens was based on the approval and guidelines of the Institutional Review Board of the Chinese PLA General Hospital.

### Cell culture

The human colon cancer cell line HCT116 was maintained in McCoy’s 5A Medium (Gibco) with 10% FBS (Gibco). The hepatocellular carcinoma cell line HepG2, gastric cancer cell line BGC823 and esophageal cancer cell line KYSE50 and colon cancer cell line SW480 were maintained in DMEM-high glucose (Gibco) with 10% FBS. HEK293T cells were maintained in DMEM-high glucose (Hyclone) with 10% FBS.

### Plasmid and constructs

The full-length sequences of MYC-AS1 were amplified from the 293T cell cDNA template with the following primers: forward 5′-CCC​AAG​CTT​GCC​TTT​TCA​TTG​TTT​TCC​A-3′ and reverse 5′-CGC​GGA​TCC​CCT​TTT​TTA​AGA​CGG​AGT​C-3′. The HuR gene was amplified with the following primers: forward 5′-CCC​AAG​CTT​ATG​TCT​AAT​GGT​TAT​GAA​GAC​C-3′ and reverse 5′-CCG​GAA​TTC​TTA​TTT​GTG​GGA​CTT​GTT​GG-3′. The amplified product of MYC-AS1 or HuR was then cloned into the expression vector pcDNA3.1.

### Transfection

HCT116 cells were plated in 6-well plates and transfected with pcDNA3.1-HuR, pcDNA3.1-MYC-AS1 or pcDNA3.1-EGFP expression plasmids using Xfect™ Transfection Reagent (631,318, Takara) according to the manufacturer’s instructions. After 48 h of transfection, cells were collected for RNA and protein analysis.

### RNA interference

HCT116 cells were plated on 6-well plates and transfected with 100 nM siRNA for 72 h using Xfect™ RNA Transfection Reagent (631,450, Takara) according to the manufacturer’s instructions. For the Dicer or HuR siRNA experiment, HCT116 cells were transfected with 100 nM siRNA for 24 h and then transfected with pcDNA3.1-MYC-AS1 or pcDNA3.1-EGFP expression plasmids using Xfect™ Transfection Reagent for an additional 48 h. All siRNA oligonucleotide duplexes used in this study were synthesized by RIBOBIO and are listed as follows.

Dicer siRNA-1: GTG​CTA​GAT​TAC​CAA​GTG​A
Dicer siRNA-2: GCA​ACT​TGG​TGG​TTC​GTT​T
Dicer siRNA-3: GGC​CGC​CTT​TCA​TAT​ATG​A
HuR siRNA-1: GAC​CCA​GGA​TGA​GTT​ACG​A
HuR siRNA-2: GAG​CGA​TCA​ACA​CGC​TGA​A
HuR siRNA-3: GGT​TGC​GTT​TAT​CCG​GTT​T
MYC-AS1 siRNA-1: GAA​AGG​TCT​CTG​GAC​AAA​AT
MYC-AS1 siRNA-2: AAA​TCA​CTC​CTT​TAG​CAA​GG
MYC-AS1 siRNA-3: CCC​AAA​GAG​CCA​CAT​CTA​AG

### Lentivirus packaging and transduction

The full-length sequences of MYC-AS1 were amplified from the 293T cell cDNA template with the following primers: forward 5′-TGC​TCT​AGA​GCC​TTT​TCA​TTG​TTT​TCC​A-3′ and reverse 5′-CGC​GGA​TCC​CCT​TTT​TTA​AGA​CGG​AGT​C-3′ and then cloned into the pCDH-CMV-MCS-EF1-Puro lentiviral vector. The *EGFP* was amplified using the following primers: forward 5′-TGC​TCT​AGA​ATG​GTG​AGC​AAG​GGC​GAG-3′ and reverse 5′-CGC​GGA​TCC​TTA​CTT​GTA​CAG​CTC​GTC-3′ and cloned into the pCDH-CMV-MCS-EF1-Puro lentiviral vector (as a control). Lentiviruses containing pCDH-CMV-MCS-EF1-Puro-MYC-AS1 or pCDH-CMV-MCS-EF1-Puro-EGFP were produced with a lentiviral packaging mix containing an optimized mixture of the three packaging plasmids, pLP1, pLP2, and pLP/VSVG, in 293T packaging cells. In brief, 293T cells were transfected with pCDH-CMV-MCS-EF1-Puro-MYC-AS1 or pCDH-CMV-MCS-EF1-Puro-EGFP and three packaging plasmids, pLP1, pLP2, and pLP/VSVG, in the presence of the Lipofectamine™ 2000 Transfection Reagent (11668019, Thermo Scientific). Viral supernatants were collected at 48 h after transfection, and viral particles were concentrated by ultracentrifugation at 50,000 g for 1.5 h at 4 °C. To establish stable cell lines expressing MYC-AS1 or EGFP, HCT116 cells were transfected with lentivirus infection for 72 h, and stable transduced clones were generated following selection with 2 μg/mL puromycin for 1–2 weeks.

### Tumor models

To assess the ability of MYC-AS1 to inhibit cell proliferation, stable HCT116 cell lines expressing MYC-AS1 or EGFP were established via lentiviral transduction. Subsequently, these cells (8 × 10^6^) were inoculated subcutaneously (*s.c.*) into the dorsal right side of nude mice. At 3, 7, 9, 11 and 14 days after inoculation, tumor sizes were measured unblinded with a caliper for calculating tumor volumes using the equation (a^2^ × b)/2 (a, width; b, length). All procedures were approved by the Animal Ethics Committee of the Chinese PLA General Hospital.

### RACE and cloning of full-length MYC-AS1

The 5′ and 3′ rapid amplification of cDNA ends (RACE) was performed using the SMARTer^®^ RACE 5′/3′ Kit (Takara, Clontech Laboratories, 634,860) following the manufacturer’s instructions. Briefly, total RNA (the genomic DNA was removed) was converted into RACE-Ready first-strand cDNA using the 5′ or 3′ CDS Primer A, provided by the kit. Next, the 5′ RACE PCRs were conducted with the 5′ gene-specific primers CCA​CAG​CAA​ACC​TCC​TCA​CAG​CCC​ACT and Universal Primer (provided by the kit) to generate the 5′ cDNA fragments. The 3′ RACE PCRs were conducted by Universal Primer and first-round 3′ gene-specific primer: GTG​TTC​GCC​TCT​TGA​CAT​TCT​CCT​CGG​TGT and then by Universal Primer and second-round 3′ gene-specific primer: AGT​GGG​CTG​TGA​GGA​GGT​TTG​CTG​TGG. The 5′ and 3′ RACE products were further characterized by 1.5% agarose gel electrophoresis and sequenced by TA cloning. After reaching the 5′ and 3′ cDNA ends, the full-length MYC-AS1 transcripts were amplified using the following primers: forward 5′-GCC​TTT​TCA​TTG​TTT​TCC​A-3′ and reverse 5′-CCT​TTT​TTA​AGA​CGG​AGT​C-3′ and subsequently cloned into the TA cloning vector for sequencing.

### Northern blot

Northern blotting assays were performed with the DIG Northern Starter Kit (cat#12039672910, Roche) according to the manufacturer’s instructions. In brief, the full-length DNA template of MYC-AS1 was first prepared by PCR amplification with the following primers: forward 5′-GCC​TCT​TGA​CAT​TCT​CCT​C-3′ and reverse 5′-TAA​TAC​GAC​TCA​CTA​TAG​GGC​GCA​ATC​AAC​CTC​CAA​CC-3′. A digoxigenin (DIG)-labeled RNA probe was then prepared using *in vitro* transcription with the T7 polymerase. Next, total RNA was separated by 2% formaldehyde gel electrophoresis at 50 V for 6 h and then transferred to positively charged nylon membranes (11209299001, Roche) by capillary transfer with 20 × SSC overnight. After RNA fixation by baking at 80 °C for 2 h, blots were hybridized with denatured DIG-labeled RNA probe overnight at 68 °C. Finally, blots were incubated with anti-digoxigenin-AP antibody (Sigma-Aldrich Cat# 11093274910, RRID:AB_2734716) for 30 min at room temperature and developed using the ready-to-use chemiluminescence substrate CDP-Star on the FluorChem Q imaging system (Protein Simple).

### Coding potential analysis of MYC-AS1 antisense RNA

Full-length MYC-AS1 was cloned into the eukaryotic expression vector pcDNA3.1 with the N-terminal start codons ATG and HA tag in all three coding possibilities, and the plasmid was subsequently transfected into HEK293T cells. P53 was cloned into the pcDNA3.1 vector and used as a positive control. After 48 h, immunoblotting was used to detect the HA tag. The primers used can be found in Table 1. Data are representative of three independent experiments.

**TABLE 1:** Primers used in this study.

Gene product	Primer sequence
c-MYC	RT primer: AAG​TTC​ATA​GGT​GAT​TGC​TCA​GGA​CAT
	Forward primer: AGCCACAGCATACATCCT
	Reverse primer: CGCACAAGAGTTCCGTAG
MYC-AS1	RT primer: GAG​ACC​TTT​CTA​ACG​TAT​TCA​TGC​CTT​GT
	Forward primer: TTC​CTC​ATC​TTC​TTG​TTC​CT
	Reverse primer: CTA​ACG​TAT​TCA​TGC​CTT​GT
GAPDH	RT primer: TGA​TCT​TGA​GGC​TGT​TGT​CAT​ACT​TCT
	Forward primer: AAG​GTG​AAG​GTC​GGA​GTC​AA
	Reverse primer: GGA​AGA​TGG​TGA​TGG​GAT​TT
LDHA	Forward primer: ATG​GCA​ACT​CTA​AAG​GAT​CAG​C
	Reverse primer: CCA​ACC​CCA​ACA​ACT​GTA​ATC​T
c-MYC sense	RT primer: GATTGCTCAGGACATTTC
	Forward primer: CACATCAGCACAACTACG
	Reverse primer: CTCAAGACTCAGCCAAGG
	Forward primer for q-PCR: AGCCACAGCATACATCCT
	Reverse primer for q-PCR: CGCACAAGAGTTCCGTAG
c-MYC antisense	RT primer: GAGGAGAATGTCAAGAGG
	Forward primer: CACATCAGCACAACTACG
	Reverse primer: CTCAAGACTCAGCCAAGG
	Forward primer for q-PCR: AGCCACAGCATACATCCT
	Reverse primer for q-PCR: CGCACAAGAGTTCCGTAG
NRAS sense	RT primer: GCT​TTC​CTT​CAA​TGG​TAG​A
	Forward primer: TGA​GAC​ATC​TAT​TCC​ACT​GA
	Reverse primer: TGT​TAT​CGG​CTC​TAT​TCT​CT
	Forward primer for q-PCR: TGA​GAC​ATC​TAT​TCC​ACT​GA
	Reverse primer for q-PCR: TGT​TAT​CGG​CTC​TAT​TCT​CT
NRAS antisense	RT primer: GAATAACTACCTCCTCAC
	Forward primer: TGA​GAC​ATC​TAT​TCC​ACT​GA
	Reverse primer: TGT​TAT​CGG​CTC​TAT​TCT​CT
	Forward primer for q-PCR: TGA​GAC​ATC​TAT​TCC​ACT​GA
	Reverse primer for q-PCR: TGT​TAT​CGG​CTC​TAT​TCT​CT
JUN sense	RT primer: GTCAAGTTCTCAAGTCTG
	Forward primer: CATCGTCATAGAAGGTCG
	Reverse primer: GGAGACAAGTGGCAGAGT
	Forward primer for q-PCR: ATCAAGGCGGAGAGGAAG
	Reverse primer for q-PCR: CACCTGTTCCCTGAGCAT
JUN antisense	RT primer: TCACCTTCTCTCTAACTG
	Forward primer: CATCGTCATAGAAGGTCG
	Reverse primer: GGAGACAAGTGGCAGAGT
	Forward primer for q-PCR: CCGCACTCTTACTTGTCG
	Reverse primer for q-PCR: CTGCTCTGGGAAGTGAGT
ETS1 sense	RT primer: AGCACTCATCGTCTGTTG
	Forward primer: TGA​TGA​TGA​TGG​CAC​AAC​T
	Reverse primer: CTGTACTTAGGCGGTGTT
	Forward primer for q-PCR: TGA​TGA​TGA​TGG​CAC​AAC​T
	Reverse primer for q-PCR: CTGTACTTAGGCGGTGTT
ETS1 antisense	RT primer: GTCGTAATAGTAGCGTAG
	Forward primer: CTATCAGACGCTCCATCC
	Reverse primer: CCGCACATAGTCCTTGAA
	Forward primer for q-PCR: CCT​CGG​ATT​ACT​TCA​TTA​GC
	Reverse primer for q-PCR: GGATGGAGCGTCTGATAG
JUNB sense	RT primer: CTTCACCTTGTCCTCCAG
	Forward primer: TCTACCACGACGACTCAT
	Reverse primer: CTGCTGAGGTTGGTGTAA
	Forward primer for q-PCR: CTA​CCA​CGA​CGA​CTC​ATA​C
	Reverse primer for q-PCR: GACAATCAGGCGTTCCAG
JUNB antisense	RT primer: GAAATCATCCTCCTCCCT
	Forward primer: TCTACCACGACGACTCAT
	Reverse primer: CTGCTGAGGTTGGTGTAA
	Forward primer for q-PCR: CTA​CCA​CGA​CGA​CTC​ATA​C
	Reverse primer for q-PCR: GACAATCAGGCGTTCCAG
FOS sense	RT primer: GAACATTCAGACCACCTC
	Forward primer: CCTTCGTCTTCACCTACC
	Reverse primer: CTATTGCCAGGAACACAG
	Forward primer for q-PCR: GACCTTATCTGTGCGTGA
	Reverse primer for q-PCR: CCTGGCTCAACATGCTAC
FOS antisense	RT primer: AGTGGAACCTGTCAAGAG
	Forward primer: CCTTCGTCTTCACCTACC
	Reverse primer: CTATTGCCAGGAACACAG
	Forward primer for q-PCR: GACCTTATCTGTGCGTGA
	Reverse primer for q-PCR: CCTGGCTCAACATGCTAC
BRAF sense	RT primer: GGT​CTC​TAA​TCA​AGT​CAT​C
	Forward primer: CCG​TTA​CAT​CTT​CTT​CCT​CT
	Reverse primer: CCT​CTT​CCT​GTG​GTA​TTG​G
	Forward primer for q-PCR: CAG​TGC​TAC​CTT​CAT​CTC​TT
	Reverse primer for q-PCR: CCT​CTC​ATC​ATC​AGT​GCT​T
BRAF antisense	RT primer: GTTGGACTATGGCTTTGT
	Forward primer: CACCACAACCTTCACCTC
	Reverse primer: TGT​CCA​AAG​AGC​AGT​TAC​C
	Forward primer for q-PCR: TCC​CAA​AGT​GCT​GAG​ATT​AC
	Reverse primer for q-PCR: CTG​AGT​GCT​GTC​CAA​AGA​G
SET sense	RT primer: CAA​GTC​ATC​ATT​AGG​AGA​G
	Forward primer: TGCTTACTATGACCTTCC
	Reverse primer: TGCTCACCATCAGACTTC
	Forward primer for q-PCR: TTCCCTCTTGTGCTCAGT
	Reverse primer for q-PCR: CTCGGTGGTGTTGATTCC
SET antisense	RT primer: CCTAATGATGACTTGAGC
	Forward primer: TGCTTACTATGACCTTCC
	Reverse primer: TGCTCACCATCAGACTTC
	Forward primer for q-PCR: CACCACCATCCAACAGAC
	Reverse primer for q-PCR: TGCTCACCATCAGACTTC
MYC-AS	aag​ctt​atg​gcc​tac​ccc​tac​gac​gtg​ccc​gac​tac​gcc​GCC​TTT​TCA​TTG​TTT​TCC​A
	aag​ctt​atg​gcc​tac​ccc​tac​gac​gtg​ccc​gac​tac​gcc​TGC​CTT​TTC​ATT​GTT​TTC​CA
	aag​ctt​atg​gcc​tac​ccc​tac​gac​gtg​ccc​gac​tac​gcc​TTG​CCT​TTT​CAT​TGT​TTT​CCA
	tct​aga​CCT​TTT​TTA​AGA​CGG​AGT​C

### Flow cytometry assay

Apoptosis was performed using the Annexin V Apoptosis Detection kit according to the manufacturer’s recommendations (BD Bioscience, Franklin). FlowJo software (RRID:SCR_008520) was used for data analysis and graphic rendering.

### Colony formation assay

HCT116 cells stably expressing MYC-AS1 or EGFP were seeded at 400 cells per well in 6-well culture plates in triplicate. After 12 days, cells were fixed with 75% ethanol for 30 min and stained with 0.2% crystal violet. The number of clones was then counted. Each experiment was repeated three times.

### Cell viability assay

HCT116 cells stably expressing MYC-AS1 or EGFP were plated into 96-well plates at a density of 2.5 × 10^3^ cells per well, and cell viability was measured by the methyl thiazolyl tetrazolium (MTT) assay (KeyGEN Biotech, Nanjing, China) at 0, 24, 48, 72, 96 and 120 h. Absorbance was measured on a microplate reader (Thermo Multiskan MK3 [Thermo Fisher Scientific, Danvers, MA, United States]) at a wavelength of 490 nm.

### DNA demethylation treatment

HCT116, HepG2, KYSE150 and BGC-823 cells were plated on 6-well plates and treated with 0, 1 or 5 μM 5-aza-2′-deoxycytidine (Aza, Sigma-Aldrich) for 48 h. The dose of Aza was selected based on preliminary studies as well as previously published studies (Cameron et al., 1999; Perez-Mancera et al., 2012). Cells were harvested after Aza treatment for RNA analysis, protein analysis and DNA methylation analysis.

### Actinomycin D treatment

HCT116 cells were plated on 6-well plates and transfected with 5 μg of pcDNA3.1-HuR or pcDNA3.1-EGFPplasmids using Xfect™ Transfection Reagent (631,318, Takara). After 72 h, cells were treated with 1 μg/mL Actinomycin D (A4262, Sigma) and harvested at 1, 2, 4 and 8 h after Actinomycin D treatment for RNA analysis.

### RNA isolation and reverse transcription

Total RNA was extracted using TRIzol^®^ Reagent (Life Technologies™, 15,596–026) according to the manufacturer’s instructions. Briefly, growth media was removed from the culture dish, and 1 mL TRIzol^®^ Reagent was added to each dish. After incubation for 5 min at room temperature, 0.2 mL of chloroform per 1 mL of TRIzol was added, and the samples were incubated for 3 min at room temperature and centrifuged at 12,000 × g for 15 min at 4 °C. The aqueous phase was removed for RNA isolation. After RNA precipitation by 100% isopropanol and RNA was washed with 75% ethanol, and the RNA pellet was resuspended in RNase-free water. The concentrations of RNA yield were measured by a spectrophotometer.

Reverse transcription was performed with PrimeScript™ RT reagent Kit with gDNA Eraser (cat#RR047B, Takara) following the manufacturer’s instructions. For strand-specific reverse transcription, the gDNA Eraser-treated RNA samples were reverse-transcribed with gene-specific primers (GSP, Table 1) at 42 °C for 15 min with PrimeScript^®^ Reverse Transcriptase.

### Quantitative PCR

Quantitative PCR (qPCR) was performed using SYBR Green Master Mix (cat# RR820B, Takara) on the CFX Connect™ Real-Time PCR Detection System (Bio-Rad). GAPDH RNA levels were used as internal control to normalize gene expression. The primers used can be found in Table 1.

### DNA extraction and bisulfite treatment

The DNeasy Blood and Tissue Kit (QIAGEN, Germany) was used to extract genomic DNA from cells or tissue samples according to the manufacturer’s instructions. The EpiTect Fast DNA Bisulfite Kit (QIAGEN, Germany) was used and 1 μg genomic DNA was used for bisulfate conversion according to the manufacturer’s instructions.

### DNA methylation pyrosequencing analysis

The forward and reverse primers used in PCR and the sequencing primers used in the pyrosequencing methylation assays were designed with PyroMark Assay Design 2.0 (QIAGEN, Germany; see Table 1). A pyrosequencing methylation analysis was conducted using the PyroMark Q24 system (QIAGEN, Germany) according to the manufacturer’s recommended protocol. Briefly, a volume of 5–20 μL of the PCR product was used for each pyrosequencing reaction based on the concentration of the PCR product and immobilized to streptavidin-coated Sepharose beads (QIAGEN, Germany). After the immobilized, PCR product was purified, denatured and washed with the PyroMark Q24 Workstation (QIAGEN, Germany). DNA strands were separated and released into a PyroMark Q24 Plate (QIAGEN, Germany). The sequencing primers were then annealed to DNA strands. The DNA methylation level was analyzed using PyroMark Q24 Advanced Software (QIAGEN, Germany). Non-CpG cytosine residues were used as controls to verify bisulfite conversion.

### Protein extraction and immunoblotting

Whole-cell lysates were prepared with Cell Lysis Buffer (10x) (Cell Signaling Technologies, 9,803) and resuspended in 4x Laemmli Sample Buffer (Bio-Rad, 161–0747) supplemented with 5% 2-mercaptoethanol. Total protein was separated by 12% SDS-PAGE at 120 V for 90 min and then transferred to polyvinylidene difluoride membranes at 50 V for 150 min. Membranes were blocked in TBS-T containing 5% nonfat dry milk (BIO-RAD). Primary antibodies were incubated overnight at 4 °C with agitation. The following antibodies were used to determine protein expression: mouse anti-c-MYC (Santa Cruz Biotechnology Cat# sc-40 AC, RRID:AB_2857941), mouse anti-GAPDH (Santa Cruz Biotechnology Cat# sc-166574, RRID:AB_2107296), mouse anti-HA (Santa Cruz Biotechnology Cat# sc-7392 HRP, RRID:AB_2894930), rabbit anti-HuR (Abcam Cat# ab200342, RRID:AB_2784506) and rabbit anti-Dicer (ab227518, Abcam, 1:1,000). After washing extensively with TBST, secondary antibodies (anti-rabbit or anti-mouse horseradish peroxidase (HRP) conjugate, 1:10,000 dilution) were incubated for 1 h at room temperature. After washing extensively with TBST, blots were developed using enhanced chemiluminescent (ECL) detection reagents on the FluorChem Q imaging system (Protein Simple).

### Immunofluorescence microscopy

Cells were fixed with 4% paraformaldehyde in phosphate-buffered saline (PBS) for 20 min at room temperature, permeabilized with 0.25% Triton X-100 for 5 min, and blocked with 2% BSA for 30 min. Cells were then incubated with rabbit anti-HuR primary antibody (ab200342, Abcam, 1:200) at room temperature for 1 h, followed by incubation with goat anti-rabbit IgG conjugated with Alexa Fluor 488 dye (Abcam Cat# ab150081, RRID: AB_2734747) at room temperature for 45 min. After five washes in PBS-T (PBS with 0.05% Tween), cells were stained with DAPI dye (Sigma, Shanghai, China) at room temperature for 10 min. Images were acquired using a Leica TCS SP8 confocal microscope, and data analysis was carried out with Leica LAS AF Lite (Leica Microsystems).

### RNA fluorescence *in situ* hybridization

RNA fluorescence *in situ* hybridization (FISH) was performed according to the manufacturer’s recommendations (RIBOBIO, Guang Zhou). We routinely ordered sets of fluorescent FISH probe mixes for MYC-AS1 from commercial sources (RIBOBIO). To achieve a sufficient signal-to-background ratio, multiple probes were targeted along each individual lncRNA sequence. A set of 15–20 probes that cover the entire length of the RNA molecule provided an optimal signal strength, and each probe carried multiple fluorophores. The pooled FISH probes were resuspended to a final concentration of 25 μM in RNase-free storage buffer and protected from light at −20 °C. Cells were fixed with 4% paraformaldehyde in phosphate-buffered saline (PBS) for 20 min at room temperature, permeabilized with 0.25% Triton X-100 for 5 min, and blocked with 2% BSA for 30 min. Cells were then incubated overnight with the MYC-AS1 probe mix at 37 °C. For colocalization studies, after RNA-FISH, cells were subjected to immunofluorescence. The pictures were captured with a Leica SP8 confocal microscope (X100) and merged.

### RNA pull-down

RNA synthesis *in vitro* transcription was performed using the T7 High Yield RNA Synthesis Kit (New England Biolabs, E2040S), and RNA purification was performed by a Monarch^®^ RNA Cleanup Kit (New England Biolabs, T2040). The 3′ terminus of an RNA strand was then biotinylated by the Pierce™ RNA 3′ End Desthiobiotinylation Kit (20,163, Thermo Scientific). In RNA pull-down experiments, we used the biotinylated MYC-AS1 and its corresponding antisense strand (the antisense RNA control) to rule out non-specific binding of MYC-AS1. The whole-cell lysate from HCT116 cells was harvested and resuspended in Pierce™ IP Lysis Buffer (87,787, Thermo Scientific) containing RNase and protease inhibitors.

RNA pull-down experiments were performed using the Pierce™ Magnetic RNA-Protein Pull-Down Kit (20,164, Thermo Scientific). Briefly, biotinylated RNA (50 pmol) was added to 50 μL of prewashed Pierce Nucleic-Acid Compatible Streptavidin Magnetic Beads and incubated for 30 min at room temperature with agitation. Next, the beads were mixed with 200 μg of the whole-cell lysate and incubated for 60 min at 4 °C with rotation. The beads were washed three times with wash buffer and then incubated for 30 min at 37 °C with elution buffer. The eluted protein samples were separated on a 12% SDS-PAGE gel and stained with a Fast Silver Stain Kit (P0017S, Beyotime) according to the manufacturer’s instructions. Protein in gel slices was digested with trypsin and identified using nano-high-performance liquid chromatography mass spectrometry (Nano-HPLC-MS) in the Proteome Research Center of Fudan University.

### RNA-binding protein immunoprecipitation (RIP)

The whole-cell lysate from HCT116 cells was harvested and resuspended in Pierce™ IP Lysis Buffer (87,787, Thermo Scientific) containing RNase and protease inhibitors. A total of 10 mg protein lysate was combined with 10 μg HuR antibody (Abcam Cat# ab200342, RRID:AB_2784506) or rabbit IgG (Abcam Cat# ab172730, RRID:AB_2687931) in 500 μL ice-cold RIP buffer and incubated overnight at 4 °C with rotation. Next, the protein sample/antibody mixture was added to a 1.5 mL microcentrifuge tube containing 50 μL prewashed Pierce Protein A/G Magnetic Beads and incubated for 1 h at 4 °C with gentle rotation. After washing, the beads were resuspended in 1 mL TRIzol RNA extraction reagent, and then the coprecipitated RNA was isolated according to the manufacturer’s instructions. Strand-specific RT-qPCR was performed to detect MYC-AS1 in the precipitates.

### Statistical analyses

Statistical analysis was performed with either Statistical Package for the Social Sciences (RRID:SCR_002865) software. Statistical significance was assessed using a two-tailed unpaired Student’s t-test with a threshold of the *p*-values <0.05.

## Results

### A set of proto-oncogenes possess antisense RNAs

**FIGURE 1:**
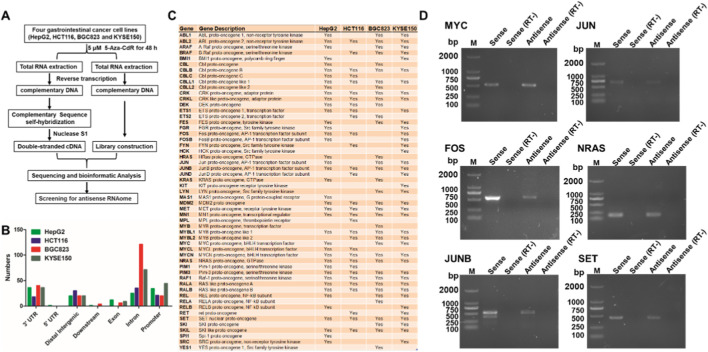
Screening for antisense RNAs of proto-oncogenes in gastrointestinal cancer cell lines. **(A)** Schematic diagram for screening the antisense RNAome. **(B)** Distribution of antisense RNAs in the gene regions of 55 proto-oncogenes. **(C)** List of 55 proto-oncogenes that possess antisense RNAs in four gastrointestinal cancer cell lines (HepG2, HCT116, BGC823 and KYSE150). **(D)** Antisense RNAs from 6 proto-oncogenes (*MYC, JUN, FOS, NRAS, JUNB* and *SET*) were validated by strand-specific RT-PCR. RT-, without reverse transcriptase. RT-was used as negative control, indicating no DNA contamination during RT-PCR.

To systematically identify endogenous antisense RNAs associated with proto-oncogenes and evaluate their potential regulatory roles in gastrointestinal cancers, we explored genome-wide antisense transcription patterns under epigenetic modulation by the DNA demethylating agent 5-Aza-CdR. The sense–antisense transcript libraries was first established from gastrointestinal cancer cell lines (KYSE50, BGC823, HCT116 and HepG2) with or without 5-Aza-CdR treatment (Figure 1A). 5-Aza-CdR treatment may facilitate potentially methylated genes to express. After high-throughput next-generation sequencing, we identified 55 eminent proto-oncogenes containing sense–antisense pairs in the region of 3′-UTRs, exon, introns or promoters, including *c-MYC, JUN, FOS, and NRAS, JUNB* and *SET* (Figures 1B,C). To confirm the existence of naturally occurring proto-oncogenes antisense RNAs, we performed RT-PCR with strand-specific primers and antisense transcripts for these proto-oncogenes were identified in the HepG2 cell line by PCR sequencing (Figure 1D). These results indicate that a set of proto-oncogenes possess antisense RNAs in human cells.

### Identification of antisense RNA MYC-AS1 from proto-oncogene *c-MYC*

**FIGURE 2:**
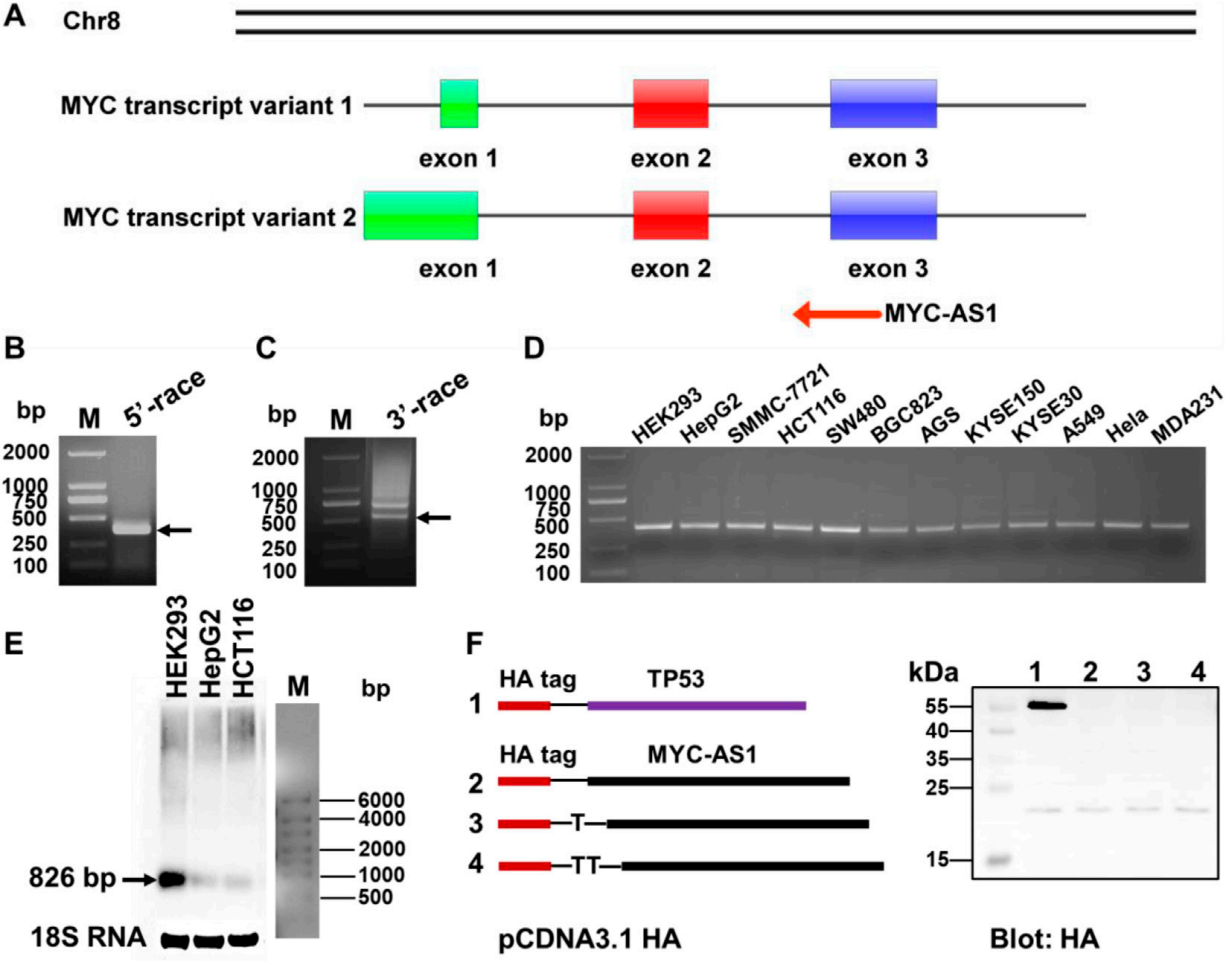
Identification of MYC-AS1 antisense RNA derived from proto-oncogene *c-MYC*. **(A)** Schematic overview of *MYC-AS1* from the proto-oncogene *c-MYC* in the human genome. **(B)** Transcriptional start site and **(C)** end site was separately identified with a 5′ cap adapter and a 3′ poly**(A)** adapter using special primers in a RACE assay that was performed with HepG2 cells treated with 5-Aza-dC. **(D)** MYC-AS1 lncRNA was detected in 11 cancer cell lines by strand-specific RT-PCR. **(E)** Northern blot analysis of *MYC-AS1* expression in HEK293, HepG2 and HCT116. **(F)** Protein-coding potential analysis of the MYC-AS1 antisense RNA. Full-length MYC-AS1 lncRNA was cloned into the eukaryotic expression vector pcDNA3.1 with an N-terminal start codon (ATG) and an HA-tag in all three reading frames, and the plasmids were subsequently transfected into HEK293 cells. After 48 h transfection, immunoblotting was used to detect the HA tag. Cells transfected using a plasmid containing TP53 sequences with an HA tag were used as a positive control.

To prioritize functionally significant antisense RNAs among the identified proto-oncogene candidates, we focused on *c-MYC* due to its central role in oncogenesis and unresolved mechanistic links to antisense regulation. By 5′ and 3′ rapid amplification of cDNA ends (RACE) analysis, we identified the *c-MYC* antisense RNA, named MYC-AS1, which is 826 bp in length (Figures 2A–C). This result was further validated by strand-specific RT-PCR and Northern blot analyses (Figures 2D,E). Additionally, we confirmed by *in vitro* translation analysis that MYC-AS1 has a poly(A) tail but does not have coding capacity (Figure 2F), indicating that it is a long noncoding RNA (lncRNA).

### MYC-AS1 inhibits cell proliferation and tumor growth

**FIGURE 3:**
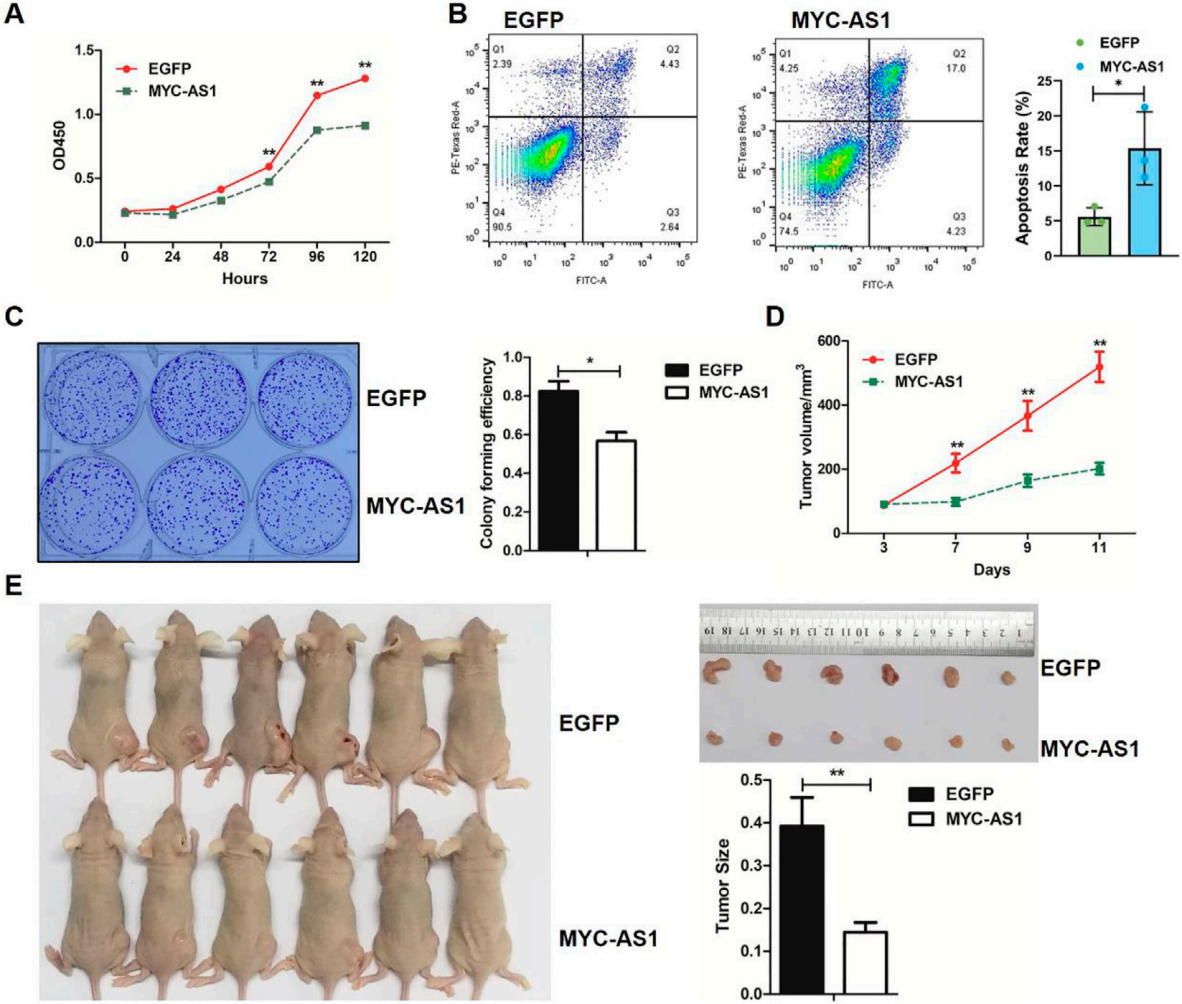
MYC-AS1 lncRNA exerts its antitumor effects by inhibiting cell proliferation and tumor growth. **(A)** MTT assay of HCT116 cells transfected with *MYC-AS1* and *EGFP*. **(B)** Apoptosis analysis of *EGFP*- or *MYC-AS1*-overexpressing HCT116 cells by flow cytometry. **(C)** Images of the colony formation assay in cells transfected with *MYC-AS1* and *EGFP*. **(D)** The growth curve of HCT116 cells transfected with *MYC-AS1* and *EGFP*. **(E)** Nude mice were xenografted with *MYC-AS1*-overexpressing and control HCT116 cells (8 × 10^6^ cells per site). Tumors were dissected and photographed after approximately 2 weeks (n = 6 per group). Tumors were weighed after removal.

To determine the tumor-suppressive potential and functional impact of MYC-AS1, we investigated its effects on cancer cell proliferation, survival, and tumorigenicity through gain-of-function assays *in vitro* and *in vivo* with a lentiviral expression vector containing the MYC-AS1 gene. Overexpression of MYC-AS1 significantly restrained proliferation and induced apoptosis in HCT116 cells (Figures 3A,B). In addition, a colony formation assay was performed to determine whether the MYC-AS1 lncRNA affects clonogenic potential. Overexpression of the MYC-AS1 lncRNA significantly decreased the number of colonies in HCT116 cells (Figure 3C). More importantly, transfection of MYC-AS1 also significantly reduced colorectal cancer cell growth and tumor weight (Figures 3D,E) in mouse xenograft models *in vivo*, suggesting that the MYC-AS1 lncRNA plays an antitumor role. Together, these data strongly suggested that MYC-AS1 lncRNA functions as a tumor suppressor to inhibit cancer proliferation.

### MYC-AS1 binds to HuR protein to enhance RNA stability

**FIGURE 4:**
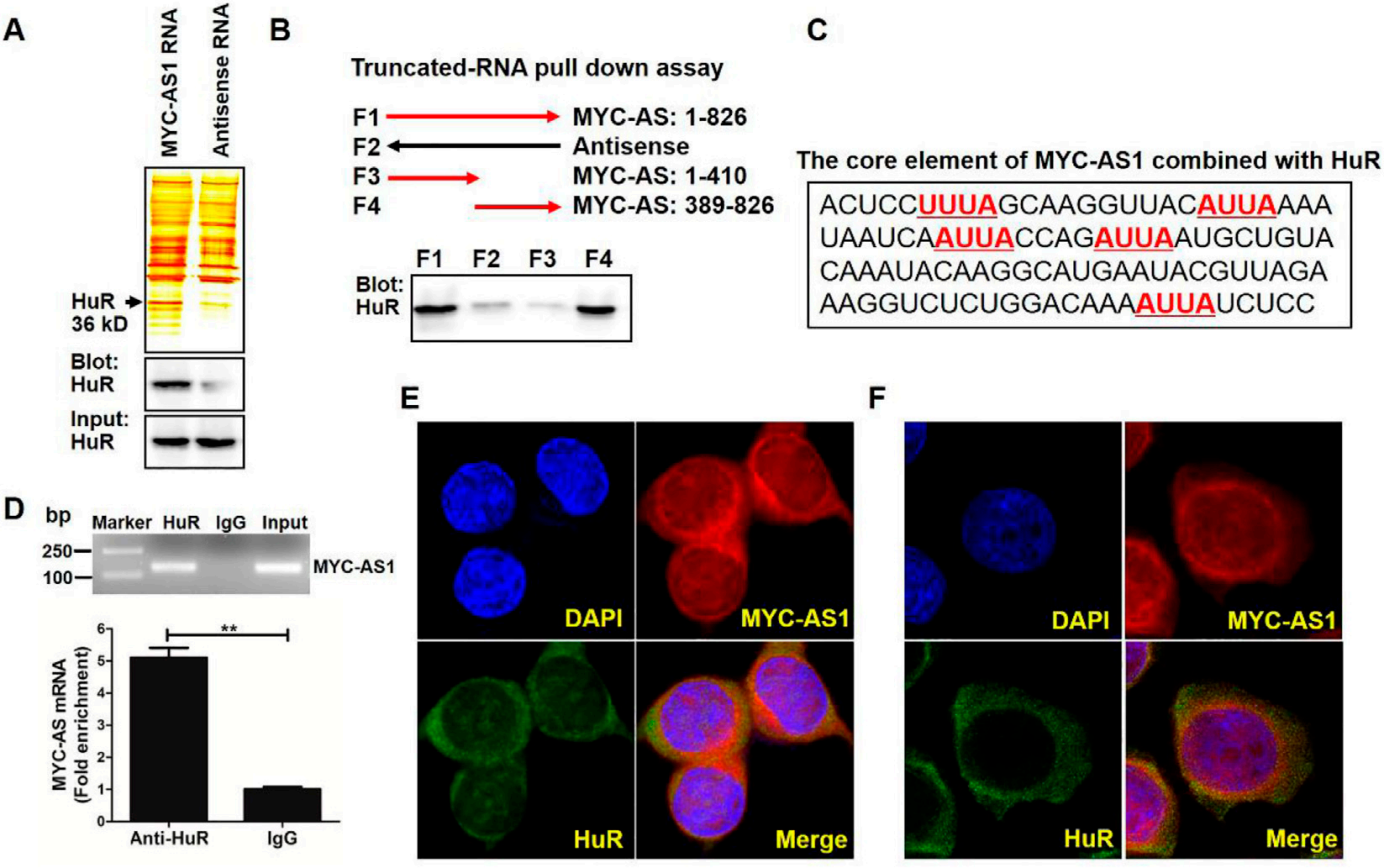
MYC-AS1 lncRNA directly binds the HuR protein in the cytoplasm. **(A)** RNA pull-down experiment was performed with MYC-AS1 and control RNAs in HCT116 cell extracts. Specific bands were identified by MS (upper panel) or immunoblotting of HuR (lower panel). **(B)** Western blot of HuR in samples pulled down by full-length (F1) or truncated MYC-AS1 lncRNA (F3: 1–410, F4: 389–826). **(C)** The 3′ fragment of MYC-AS1 RNA (436–538nt) contains four ARE motif (AUUA), which is a core element combined with HuR. **(D)** RT-PCR and RT-qPCR detection of MYC-AS1 RNA precipitated with the HuR-specific antibody in a RIP assay with HCT116 cell extracts. **(E)** RNA FISH assay of MYC-AS1 RNA combined with immunofluorescence detection of HuR was performed in HCT116 cells. **(F)** RNA FISH assay of MYC-AS1 RNA combined with immunofluorescence detection of HuR was performed in BGC-823 cells. DAPI, 4′,6-diamidino-2-phenylindole.

To uncover the molecular mechanism by which MYC-AS1 exerts its tumor-suppressive function, we sought to identify RNA-binding proteins interacting with MYC-AS1 and elucidate their roles in mediating its biological activity. Firstly, we performed a pulldown assay with biotinylated MYC-AS1 RNA in combination with mass spectrometry (MS) to search for potential MYC-AS1 RNA-interacting proteins. Human antigen R (HuR, also called ELAV-like protein 1), a regulator of mRNA stability of the c-MYC proto-oncogene, was identified (Lafon et al., 1998; Ma et al., 1997). Western blot analysis validated that HuR also specifically binds to MYC-AS1 RNA (Figure 4A). Truncated RNA pulldown assays and bioinformatic analysis indicated that the 3′ fragment of MYC-AS1 RNA contains an AU-rich element (ARE) (AUUA), which is a core element for binding with HuR (Figures 4B,C). RNA-binding protein immunoprecipitation assay (RIP) further verified the specificity of this interaction (Figure 4D). Moreover, RNA FISH results confirmed that MYC-AS1 RNA is colocalized with HuR in the cytoplasm but not in the nucleus of HCT116 and BGC-823 cells (Figures 4E,F).

### MYC-AS1 lncRNA modulates *c-MYC* expression

**FIGURE 5:**
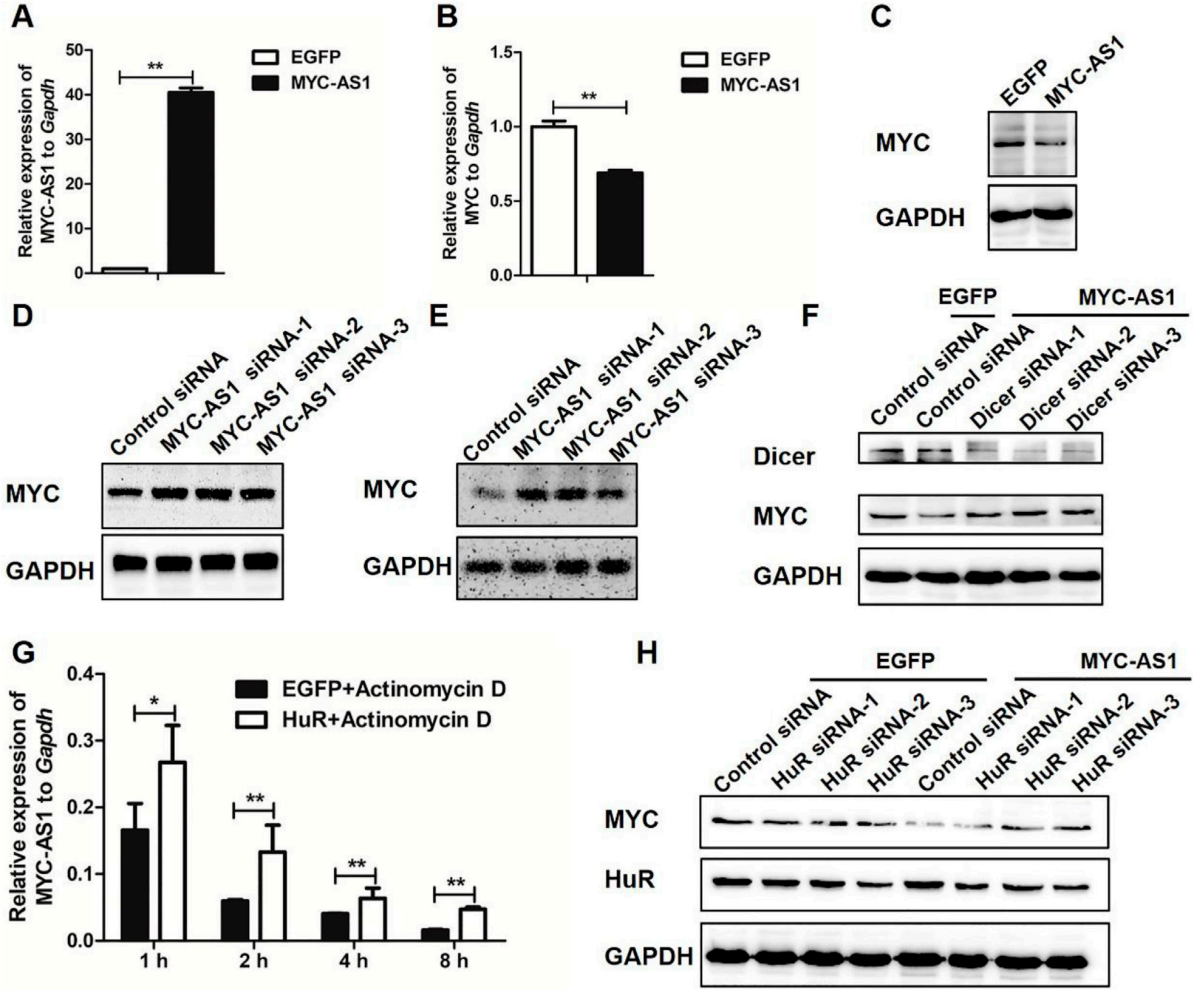
MYC-AS1 lncRNA modulate *c-MYC* expression. **(A)**RT-qPCR detection of *MYC-AS1* in HCT116 cells with a lentivirus-mediated expression system containing *MYC-AS1* or *EGFP*. **(B)** RT-qPCR detection of *c-MYC* in HCT116 cells with a lentivirus-mediated expression system containing *MYC-AS1* or *EGFP*. **(C)** Immunoblot of c-MYC protein levels in HCT116 cells with a lentivirus-mediated expression system containing *MYC-AS1* or *EGFP*. **(D)** Immunoblot of c-MYC protein levels after *MYC-AS1* knockdown with siRNA in HCT116 cells. **(E)** Immunoblot of c-MYC protein levels after *MYC-AS1* knockdown with siRNA in HEK293 cells. **(F)** *DICER* knockdown impairs MYC-AS1 RNA repression effect on *c-MYC*. Western blot results indicate that MYC-AS1 RNA repression effect on *c-MYC* may require DICER, suggesting that MYC-AS1 lncRNA may represses *c-MYC* by an RNAi mechanism. **(G)** MYC-AS1 RNA binding to HuR increases MYC-AS1 RNA stability. RNA stability assays were performed in HCT116 cells using actinomycin D to disrupt RNA synthesis, and the degradation rate of MYC-AS1 RNA was measured and calculated over 8 h. HCT116 cells were first transfected with HuR or EGFP for 72 h and were then treated with 1 μg/mL actinomycin D for 1∼8 h. **(H)** HuR knockdown affects MYC-AS1 lncRNA repression effect on *c-MYC*. Western blot results show that HuR protein is involved in the regulation of MYC-AS1 RNA on *c-MYC*. *P < 0.05 and **P < 0.01 (two-tailed Student’s t-test).

To determine the regulatory effect of MYC-AS1 on the proto-oncogene c-MYC and elucidate the molecular mechanism underlying this interaction, we investigated whether MYC-AS1 modulates c-MYC expression through RNA interference (RNAi) pathways and HuR-mediated stabilization. We found that overexpression of *MYC-AS1* significantly inhibited the expression of c-MYC mRNA and protein in HCT116 cells (Figures 5A–C). Inversely, knockdown of *MYC-AS1* significantly increased the expression of *c-MYC* in both HCT116 and HEK293 cells (Figures 5D,E). To verify the molecular mechanism by which the oncogene *c*-*MYC* is regulated by the antisense MYC-AS1 lncRNA, the DICER gene was knocked down by RNAi. Reduced DICER can abolish the inhibitory effect of MYC-AS1 lncRNA on *c*-*MYC* (Figure 5F), indicating that MYC-AS1 lncRNA inhibits the expression of *c*-*MYC* through an RNAi mechanism. We also found that HuR overexpression inhibits the actinomycin D-mediated degradation of MYC-AS1 (Figure 5G). Furthermore, the inhibitory effect of MYC-AS1 lncRNA on *c-MYC* expression was weakened when HuR was disturbed. HuR knockdown not only affects *c-MYC* expression but weakened the MYC-AS1 lncRNA repression effect on c-MYC (Figure 5H). These results together indicate that MYC-AS1 lncRNA can bind HuR protein to enhance the stability of MYC-AS1 lncRNA, which is beneficial for inhibiting *c*-*MYC* expression.

### MYC-AS1 lncRNA inhibits expression of c-MYC-targeted gene LDHA

**FIGURE 6:**
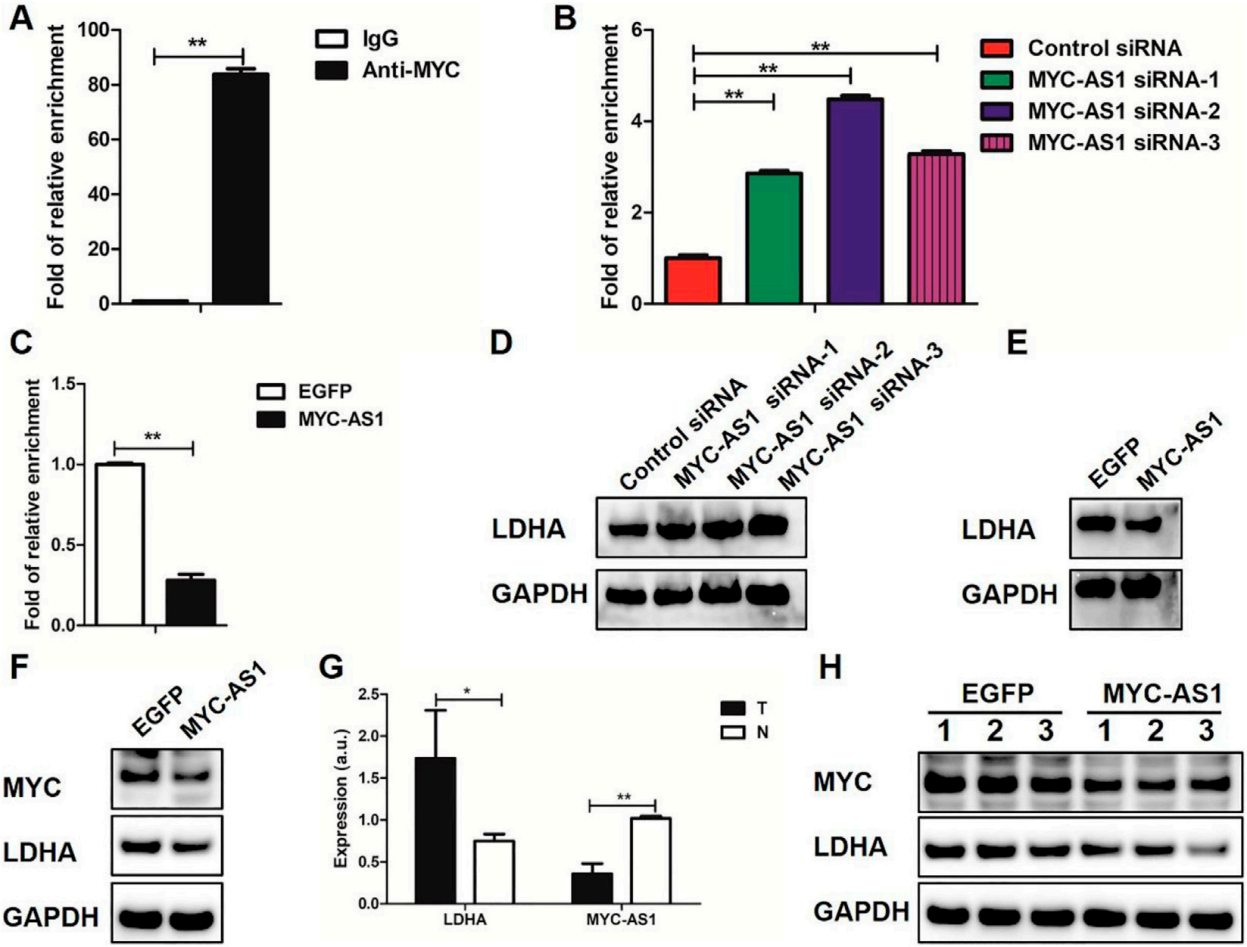
MYC-AS1 lncRNA regulates *LDHA* expression through c-MYC. **(A)** LDHA DNA was detected in the chromatin sample immunoprecipitated from HCT116 cells using an antibody against c-MYC; **(B,C)** Real-time PCR of the ChIP samples shows the binding efficiency of c-MYC to the LDHA gene promoter; **(D)** Immunoblot of proteins levels in HCT116 cells transfected with siRNAs; **(E)** Immunoblot of proteins levels in HCT116 cells with a lentivirus-mediated expression system containing *MYC-AS1* or *EGFP*; **(F)** Immunoblot of proteins levels in SW480 cells with a lentivirus-mediated expression system containing *MYC-AS1* or *EGFP*; **(G)** RT-qPCR detection of *LDHA* and *MYC-AS1* in colorectal cancer tissues. “T” stands for tumor while “N” stands for normal. **(H)** Immunoblot of proteins levels in the nude mice, which were xenografted with MYC-AS1-overexpressing and control HCT116 cells.

To determine whether MYC-AS1-mediated suppression of c-MYC transcriptionally regulates downstream oncogenic targets such as LDHA, and to validate the clinical relevance of this regulatory axis, we investigated the functional interplay between MYC-AS1, c-MYC, and LDHA across cellular models, patient samples, and *in vivo* xenografts. We firstly performed ChIP coupled with qPCR (ChIP-qPCR) for c-MYC in HCT116 cells. The binding of c-MYC to the LDHA gene was verified in HCT116 cells (Figure 6A). Moreover, the binding of c-MYC to the LDHA gene was increased by MYC-AS1 knockdown (Figure 6B) but reduced by overexpression of MYC-AS1 (Figure 6C). Western blot data further showed that *LDHA* expression was significantly increased in HCT116 cells transfected with MYC-AS1 siRNAs (Figure 6D). However, overexpression of MYC-AS1 significantly reduced the expression of the LDHA gene in HCT116 cells (Figure 6E). MYC-AS1 overexpression also significantly inhibited the expression of the MYC and LDHA gene in SW480 cells (Figure 6F). These results together indicate that MYC-AS1 lncRNA may negatively regulate LDHA transcription via c-MYC in colorectal cancer cells. Interestingly, the samples with lower MYC-AS1 expression displayed strong expression of *LDHA*, while samples with high MYC-AS1 expression showed low levels of *LDHA* (Figure 6G). We also confirmed that overexpression of MYC-AS1 significantly inhibited the expression of MYC and LDHA in the subcutaneous model xenografts experiments (Figure 6H). These data indicate that MYC-AS1 expression is negatively correlated with *c-MYC* and *LDHA* expression in CRC tissues.

### Epigenetic silencing of MYC-AS1 contributes to *c-MYC* activation

**FIGURE 7:**
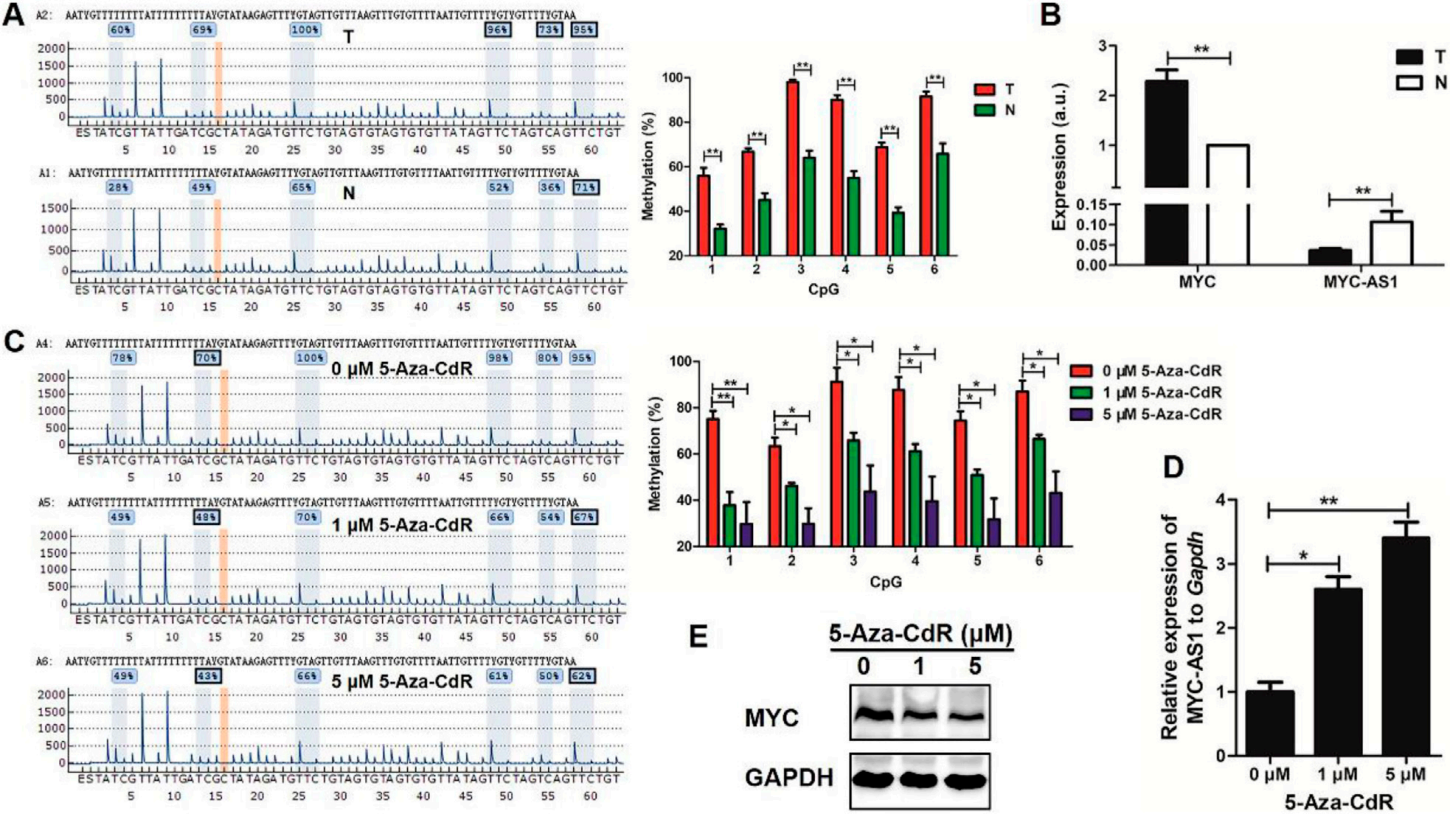
A significant inverse correlation exists between the expression levels of *MYC-AS1* and its corresponding proto-oncogene *c-MYC* in colorectal cancer. **(A)** Representative pyrograms and methylation levels for each CpG site in the *MYC-AS1* region in colorectal cancer tissues. **(B)** RT-qPCR detection of *c-MYC* and *MYC-AS1* in colorectal cancer tissues (n = 24 samples). **(C)** Representative pyrograms and methylation levels for each CpG site in the *MYC-AS1* region in HCT116 colorectal cancer cells treated with 0, 1 and 5 *μ*M 5-Aza-CdR. **(D)** RT-qPCR detection of *MYC-AS1* in HCT116 cells. **(E)** Immunoblot analysis of c-MYC in HCT116 cells treated with 0, 1 or 5 *μ*M 5-Aza-CdR.

To investigate the epigenetic regulation of MYC-AS1 in colorectal cancer and its functional consequences on oncogene activation, we examined the DNA methylation status of MYC-AS1 in clinical tissues and evaluated whether demethylation therapy (5-Aza-CdR) could restore its expression to suppress c-MYC-driven oncogenicity. We found that the expression of MYC-AS1 was inversely correlated with its DNA methylation in clinical colon cancer tissues and cell lines by bisulfite-PCR-pyrosequencing and RT-qPCR analyses. The MYC-AS1 gene was hypermethylated in cancer tissues but hypomethylated in matched normal tissues (Figure 7A). Correspondingly, MYC-AS1 expression was much lower in cancer tissues than in matched normal tissues, while the expression of the oncogene *c*-*MYC* shows the opposite trend with higher expression in tumor tissues than in the matched normal tissues (Figure 7B). When the HCT116 cells were treated with 5-Aza-CdR, the expression of MYC-AS1 was significantly induced, but the expression of the c-MYC gene was inhibited at both the mRNA and protein levels (Figures 7C–E). These results demonstrate that demethylation of the MYC-AS1 gene can restore the expression of the MYC-AS1 antisense lncRNA, which further represses *c-MYC* expression. These results indicate that the high-level expression or activation of the oncogene is attributed to epigenetic silencing of their counterpart antisense RNA by DNA hypermethylation and that DNA methylation inhibitor can restore antisense RNA transcription, which further represses oncogene expression.

## Discussion

The present study unveils a previously uncharacterized mechanism of c-MYC deregulation in colon cancer, driven by the epigenetic silencing of its antisense lncRNA, MYC-AS1. This event removes a critical repressor of c-MYC, culminating in its aberrant activation and enhanced tumor proliferation. This MYC-AS1/c-MYC axis constitutes a pivotal epigenetic vulnerability in this malignancy, highlighting its potential as a therapeutic target.

Our findings demonstrate that epigenetic silencing of antisense RNAs results in derepression or activation of proto-oncogenes. The activation of antisense RNAs may be an effective and safe strategy to inhibit the expression of proto-oncogenes. In particular, we discover that a set of proto-oncogenes possess antisense RNAs and that widespread activation of oncogenes may be attributed to epigenetic silencing of their counterpart antisense lncRNAs in multiple types of cancers. This finding enriches the understanding of epigenetic mechanisms underlying oncogene activation in tumorigenesis.

There has been an ongoing and endless debate about the molecular mechanisms underlying the clinical efficacy of the DNA methylation inhibitors in the treatment of cancers and whether this efficacy is a direct consequence of its effect on global demethylation (Issa and Kantarjian, 2009). A recognized antitumor mechanism of 5-Aza-CdR is the reactivation of aberrantly methylated TSGs by demethylation (Navada et al., 2014). Consistent with this observation, many TSG promoters are hypermethylated in cancer cells (De Carvalho et al., 2012). Another mechanism is that 5-Aza-CdR activates anticancer innate immunity through transcriptional activation of endogenous retroviral sequences (Chiappinelli et al., 2015; Licht, 2015; Roulois et al., 2015). The present study might be indicated that 5-Aza-CdR impedes rather than promotes the expression of proto-oncogenes via epigenetic activation of antisense RNAs.

Our study verifies this phenomenon by focusing on the proto-oncogene *c*-*MYC*. c-MYC contributes to initiate and maintain tumorigenesis in many human cancers and regulates the antitumor immune response through CD47 and PD-L1 (Casey et al., 2016), providing new insights into immunotherapies and epigenetic therapies (Casey et al., 2016; Dang, 2012; Stine et al., 2015; Topper et al., 2020). Although previous studies have reported that the MYC gene can be transcribed bidirectionally in humans, mice, rodents and bovines (Bentley and Groudine, 1986; Dean et al., 1983; Kindy et al., 1987; Spicer and Sonenshein, 1992), we identified MYC-AS1 as a novel antisense noncoding RNA that is completely different from those previously reported (Napoli et al., 2017). MYC-AS1 lncRNA functions as an anticancer molecule mainly through inhibition of the sense c-MYC gene via the RNAi pathway. Additionally, MYC-AS1 lncRNA strengthens its antitumor function by binding to HuR to enhance itself stability. The HuR protein contains several RNA recognition motifs and selectively binds the ARE motif, which is a well-studied signal present in the 3′-UTRs of many clinically relevant mRNAs, including those of cytokines, oncogenes and growth factors (Vasudevan and Steitz, 2007). The 3′ fragment of MYC-AS1 RNA contains an ARE motif (i.e., AUUA), which is a core element for binding with HuR. The stability of MYC-AS1 lncRNA is enhanced by binding to HuR, and thus is beneficial for the inhibition of *c-MYC* expression through the RNAi mechanism. In addition, HuR is involved in 3′-UTR ARE-mediated c-MYC mRNA stabilization (Lafon et al., 1998; Ma et al., 1997) and regulates the expression of *c-MYC* (Liu et al., 2015; Liu et al., 2009). MYC-AS1 lncRNA may negatively regulate *c-MYC* by competitively inhibiting the binding of HuR to the 3′-UTR of the c-MYC mRNA.

Our finding that MYC-AS1 acts as a tumor suppressor contrasts with the oncogenic role of PVT1, another non-coding RNA in the same genomic locus as MYC. PVT1 is frequently co-amplified with MYC in cancers and promotes MYC protein stability and activity (Tseng et al., 2014). In contrast, MYC-AS1 is epigenetically silenced in tumors and represses MYC mRNA abundance through an RNAi-like mechanism. This divergence, where one antisense transcript enhances MYC-driven oncogenesis and the other suppresses it, reveals a complex layer of MYC regulation and establishes MYC-AS1 as a distinct functional entity. The mechanism we describe, epigenetic silencing of a trans-acting antisense lncRNA leading to oncogene activation, may extend beyond MYC. Numerous key oncogenes, such as EGFR, KRAS, and CCND1, harbor uncharacterized antisense transcripts (Mercer and Mattick, 2013; Katayama et al., 2005). Hypermethylation-mediated silencing of such natural antisense RNAs could represent a widespread yet overlooked pathway contributing to oncogene activation in cancer. Systematic study of these antisense partners may thus uncover a new class of epigenetically regulated therapeutic targets.

Therapeutically, reactivating MYC-AS1 represents a promising strategy for MYC-driven cancers. However, current DNA methyltransferase inhibitors (e.g., azacitidine, decitabine) pose a significant caveat: although they may restore MYC-AS1 expression, their genome-wide demethylation risks activating promiscuous promoters, transposable elements, and dormant oncogenes, potentially causing genomic instability (Kelly et al., 2010). This highlights the need for precision epigenetic targeting. Future approaches could employ CRISPR/dCas9 systems engineered to recruit demethylating activity specifically to the MYC-AS1 promoter. Such locus-specific intervention would aim to suppress MYC without off-target epigenetic effects, offering a safer and more precise therapeutic route.

**FIGURE 8:**
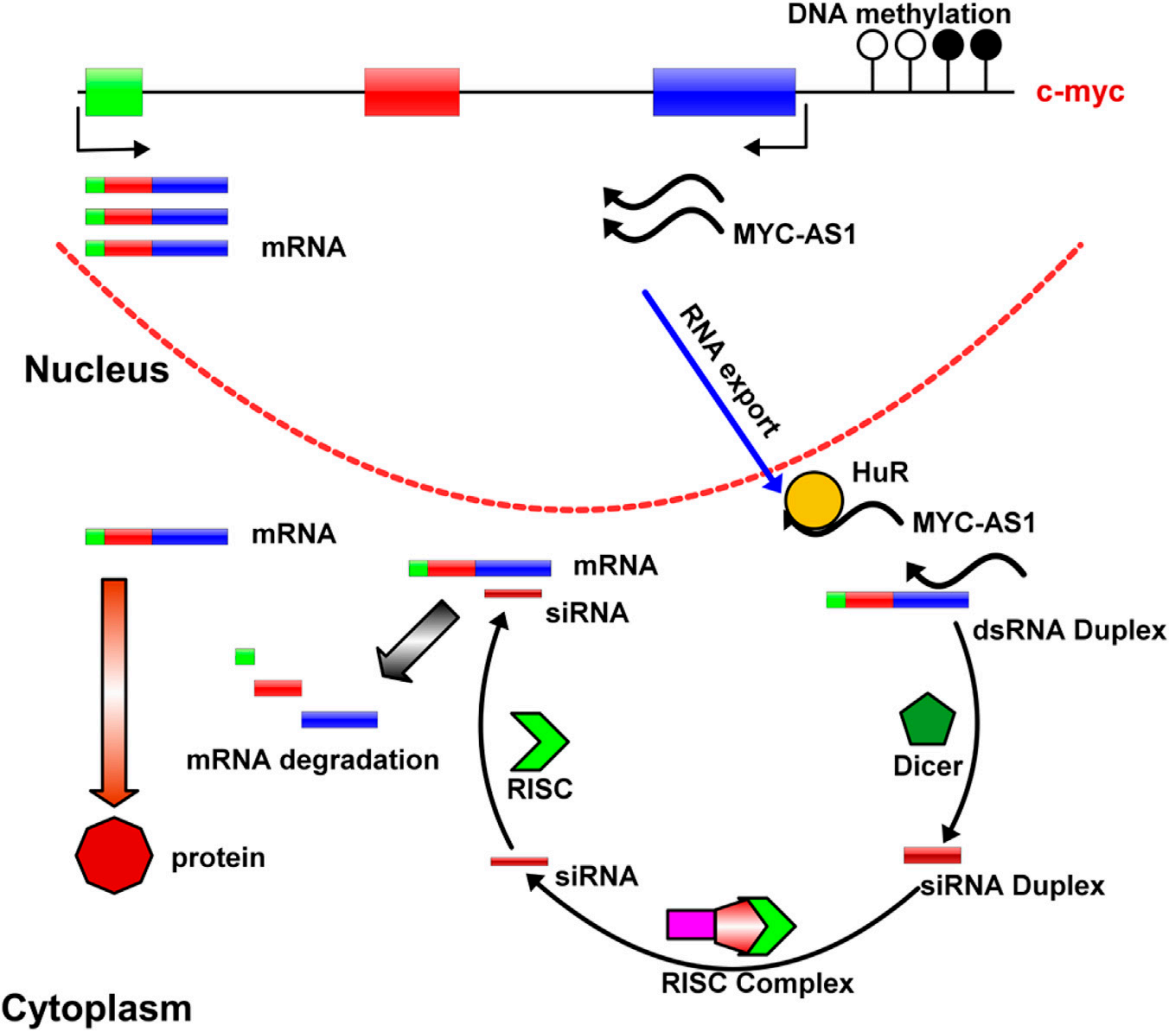
A model of *MYC-AS1* anti-cancer mechanism. The MYC-AS1 lncRNA is exported into the cytoplasm, where it can form RISC complex with other molecules and give rise to c-MYC RNA depredation. In addition, MYC-AS1 RNA can bind HuR protein by competing with c-MYC RNA and shorten c-MYC RNA half-life.

In summary, we find that epigenetic silencing of the MYC-AS1antisense lncRNA directly contributes to activation or upregulation of the sense c-MYC gene. MYC-AS1 lncRNA clearly functions as a tumor suppressor that strictly controls *c*-*MYC* expression. Once this suppressor becomes defective due to events such as epigenetic silencing, the *c*-*MYC* oncogene is abnormally activated. These results reveal a novel mechanism of tumorigenesis by which oncogenes are activated by epigenetic silencing of their counterpart antisense lncRNAs (the working model is shown in Figure 8). Further investigation remains to determine whether antisense RNAs for other proto-oncogene perform their functions in a similar manner. MYC-AS1, a specific regulator of tumorigenesis, may facilitate the design and development of new anticancer drugs.

## Data Availability

The data presented in the study are deposited in the NCBI repository, accession number PRJNA1166350.
